# Australian guideline on wound classification of diabetes-related foot ulcers: part of the 2021 Australian evidence-based guidelines for diabetes-related foot disease

**DOI:** 10.1186/s13047-021-00503-6

**Published:** 2021-12-03

**Authors:** Emma J. Hamilton, Joanna Scheepers, Hayley Ryan, Byron M. Perrin, James Charles, Jane Cheney, Stephen M. Twigg, on behalf of the Australian Diabetes-related Foot Disease Guidelines & Pathways Project

**Affiliations:** 1 Department of Endocrinology and Diabetes, Fiona Stanley Hospital, Murdoch, Australia; 2 University of Western Australia, Medical School, Fiona Stanley Hospital, Murdoch, Australia; 3 St John of God Midland Public and Private Hospitals, Midland, WA Australia; 4 Wounds Australia Limited, WoundRescue Pty Ltd, Canberra, ACT Australia; 5 La Trobe Rural Health School, La Trobe University, Bendigo, Australia; 6 National Indigenous Knowledge’s, Education, Research and Innovation Institute, Faculty of Arts and Education, Deakin University, Geelong, Australia; 7 Diabetes Victoria, Melbourne, Australia; 8 Department of Endocrinology, Royal Prince Alfred Hospital, Camperdown, Australia; 9 University of Sydney, Sydney Medical School, Faculty of Medicine and Health, Sydney, NSW 2006 Australia; 10 Diabetes Feet Australia, Brisbane, Australia; 11 Australian Diabetes Society, Sydney, Australia

**Keywords:** Wound classification, Guidelines, Adapt, Adopt, Diabetes-related foot ulcers

## Abstract

**Background:**

Wound classification systems are useful tools to characterise diabetes-related foot ulcers (DFU) and are utilised for the purpose of clinical assessment, to promote effective communication between health professionals, and to support clinical audit and benchmarking. Australian guidelines regarding wound classification in patients with DFU are outdated. We aimed to adapt existing international guidelines for wound classification to develop new evidence-based Australian guidelines for wound classification in people with diabetes and DFU.

**Methods:**

Recommended NHRMC procedures were followed to adapt suitable International Working Group on the Diabetic Foot (IWGDF) guidelines on wound classification to the Australian health context. Five IWGDF wound classification recommendations were evaluated and assessed according to the ADAPTE and GRADE systems. We compared our judgements with IWGDF judgements to decide if recommendations should be adopted, adapted or excluded in an Australian context. We re-evaluated the quality of evidence and strength of recommendation ratings, provided justifications for the recommendation and outlined any special considerations for implementation, subgroups, monitoring and future research in an Australian setting.

**Results:**

After the five recommendations from the IWGDF 2019 guidelines on the classification of DFUs were evaluated by the panel, two were adopted and three were adapted to be more suitable for Australia. The main reasons for adapting, were to align the recommendations to existing Australian standards of care, especially in specialist settings, to maintain consistency with existing recommendations for documentation, audit and benchmarking and to be more appropriate, acceptable and applicable to an Australian context. In Australia, we recommend the use of the SINBAD system as a minimum standard to document the characteristics of a DFU for the purposes of communication among health professionals and for regional/national/international audit. In contrast to the IWGDF who recommend against usage, in Australia we recommend caution in the use of existing wound classification systems to provide an individual prognosis for a person with diabetes and a foot ulcer.

**Conclusions:**

We have developed new guidelines for wound classification for people with diabetes and a foot ulcer that are appropriate and applicable for use across diverse care settings and geographical locations in Australia.

**Supplementary Information:**

The online version contains supplementary material available at 10.1186/s13047-021-00503-6.

## Background

One Australian loses a limb, or part thereof, every 2 h as a consequence of diabetes-related foot disease (DFD) [1, 2]. DFD is defined as ulceration, infection, ischaemia or neuroarthropathy of the foot in a person with diabetes [3–6]. The annual burden of DFD in Australia is high with an estimated 27,600 public hospitalisations, 4400 lower limb amputations, 1700 deaths, and health system costs of $1.6 billion each year [3, 4, 7–9]. Management of DFD by interdisciplinary high risk foot services (iHRFS) increases the percentage of healed ulcers, and reduces wound healing times, amputations and hospitalisations [10]. Effective assessment, documentation and communication of clinical information and audit of patient outcomes is central to acheiving optimal outcomes for patients living with DFD. Aboriginal and Torres Strait Islander Australians have a 38-fold elevated risk of developing DFD, including diabetes-related peripheral neuropathy, DFU and amputation [11, 12]. Foot health complications in Aboriginal and Torres Strait islander communities is historic [13, 14] and this also impacts on social and emotional well-being[15]. This relates to outcome 1 in the new 2020 Closing the Gap in Partnership agreement “Aboriginal and Torres Strait Islander people enjoying long and healthy lives” with potential avoidable mortality rates and rates of accessing health services. These recommendations also address outcome 14 “Aboriginal and Torres Strait Islander people enjoying high levels of social and emotional wellbeing” in relation to “psychological distress” caused by hospitalisation due to ulceration and amputation [16]. Therefore, implementing strategies for the prevention of DFU is critical to all Australians, and will likely contribute to lowering the national health care burden [17]. To a greater extent, addressing health disparities experienced by Aboriginal and Torres Strait Islander people is also paramount [11].

A diabetes-related foot ulcer (DFU) is a break in the skin of the foot in a person with diabetes which does not promptly heal [18]. DFUs may vary in regard to precipitant, characteristics such as location, size and depth and there are a number of different factors which may influence DFU outcomes, such as healing time and risk of lower extremity amputation (LEA) [18, 19]. Wound classification systems are useful tools to support clinical assessment, aid effective communication between health professionals, assist with timely triage of referrals to specialist services such as iHRFS, and to guide clinical decision making and prognosis in certain settings, as well as support clinical audit and benchmarking [18, 19].

There have been a number of review articles of DFU classification systems, including a recent critical review by the IWGDF [18, 20–23]. DFU classification systems vary in their intended purpose and clinical use, may be predominantly descriptive or generate a score or risk level, be simple relying on clinical examination findings, or complex requiring specialised equipment or expertise [18, 19]. A DFU classification system intended to provide a risk assessment or prognosis for an individual patient will require more detailed information and evaluation compared with a DFU classification system designed for the comparison of outcomes between populations, the latter which would ideally be simple, quick and require no specialised equipment [19]. In total, 37 DFU classification systems were identified by the IWGDF, of which 19 were reviewed in detail, suggesting it is likely no one DFU classification system is ideally suited for all clinical purposes and populations [18, 19].

Based on existing evidence from review of clinical cohorts and consensus expert opinion the IWGDF determined there were eight key factors which were most important for predicting DFU outcomes, namely: patient factors (end-stage renal disease); limb factors (presence of peripheral arterial disease, loss of protective sensation); and ulcer factors (area, depth, location, number of ulcers and presence of infection); however no one DFU classification system currently comprises all of the aforementioned parameters [18, 19, 24–30]. In the 2019 IWGDF DFU classification guidelines five key questions or clinical scenarios were identified and considered to be of critical importance regarding the use of wound classification systems in people with a DFU: a) the most appropriate DFU classification system for the purposes of communication among health professionals b) for the purpose of providing a prognosis for DFU outcomes c) for guiding clinical management of DFU complicated by infection d) for guiding decision making regarding benefit from revascularisation in a patient with DFU, and e) for the purpose of regional/national/international audits [18, 19].

National evidence-based Australian guidelines for prevention, identification and management of foot complications in diabetes were last published in 2011 and are now outdated [31]. As there are existing international DFD guidelines that were recently updated and suitable for adaptation to an Australian context, here we present the new Australian evidence-based guideline for wound classification in people with DFU, adapted from recent 2019 guidelines from the IWGDF [19, 32, 33].

## Methods

The process for development of these guidelines has been overseen by the Australian DFD guidelines working group and is described in detail in an accompanying guidelines development paper [33]. NHMRC recommendations for adapting source guidelines were followed, which recommend an approach using eight steps: i) defining the scope; ii) identifying potential source guidelines; iii) assessing the suitability of source guidelines; iv) assessing and deciding which source guideline recommendations to adopt, adapt, or exclude; v) drafting new recommendations and rationale for the context; vi) collating recommendations and rationale into new guidelines; vii) developing clinical pathway(s) to aide implementation; and viii) consultation and endorsement of the final guidelines [33–36]. The Australian DFD guidelines development paper reports the findings of the first three steps and concludes that the 2019 International Working Group on the Diabetic Foot (IWGDF) guidelines were the only suitable international source guidelines to adapt for this new guideline [33]. The subsequent steps are described in this manuscript.

A national expert panel was established by the Australian DFD Guidelines working group to develop this wound classification guideline, consisting of recognised multi-disciplinary experts in DFU management and wound classification, along with a consumer representative and Aboriginal and Torres Strait Islander DFD experts [33]. The panel members were provided with the recommendations from the 2019 guidelines on the classification of diabetic foot ulcers and supporting critical review from the IWGDF [18, 19, 33].

Two members of the Wound Classification panel independently screened and reviewed each of the five IWGDF DFU classification recommendations (and rationale) for the quality of evidence, strength of recommendation, acceptability and applicability in an Australian context, using a customised 7-item ADAPTE evaluation form [33, 35]. The panel subsequently met to review and discuss ratings for all five recommendations until consensus decisions for all recommendations were reached. The panel were empowered to realise a consensus decision for each IWGDF recommendation. If the panel agreed with the quality of evidence and strength of a recommendation made by IWGDF, and found it acceptable and applicable in the Australian context, then the recommendation was adopted. Any recommendations where the panel determined uncertainty or disagreement existed with: the quality of evidence; strength of recommendation; or acceptability or applicability in the Australia context, were referred to be fully assessed in the next stage, of full assessment [33, 35].

Recommendations requiring full assessment used a customised GRADE Evidence to Decision (EtD) tool [33, 36–38]. One panel member extracted and populated the EtD tool, with relevant supporting evidence text for the recommendation from the IWGDF guidelines on the classification of diabetic foot ulcers, and critical review plus any more recent relevant published material [18, 19]. The EtD tool describes eight important criteria: the problem, desirable effects, undesirable effects, quality (or certainty) of evidence, values (of importance of outcomes), balance of effects, acceptability, and applicability [33, 36–38]. The populated EtD tool was checked by a second panel member and any disagreements were discussed until consensus was reached. The panel met to discuss and gain consensus on their summary judgements for the eight criteria [37, 38], and compared their judgements with the IWGDF [33, 36].

Based on the level of agreement between the panel and IWGDF summary judgements, the panel then discussed and made a consensus decision on adopting, adapting, or excluding the recommendation concerned for the Australian national context [33, 36]. These decisions were defined as follows: adopted, if there were no substantial differences between the panel and IWGDF summary judgements; adapted, if there were substantial differences; and excluded, if there were substantial differences and/or the panel concluded the recommendation was not acceptable or applicable in Australia [33, 36]. Any disagreements within the panel were discussed until consensus was reached.

Those recommendations the panel decided to adapt had their quality of evidence, strength of recommendation rating [36, 37, 39] and written recommendations re-evaluated, via consensus based on the panel’s summary judgements [33, 36]. The panel rated the quality of evidence as per the GRADE system as: high, if the panel was very confident that the findings from the supporting evidence were from studies with low risk of bias that reported consistent effects and further research was unlikely to change that confidence; moderate, if moderate confidence in the risk of bias or consistency of effects and further research was likely to impact that confidence further; low, if limited confidence in the risk of bias and inconsistency of effects and further research was very likely to impact confidence; and very low, if very little confidence in the available supporting evidence [37, 39]. The panel rated the strength of recommendations also based on GRADE system by weighing up the balance of effects, quality of evidence, values, applicability and acceptability [37, 39] in the Australian national context [33] as: strong, if there was clearly a moderate-to-large difference in the balance of effects between the intervention compared with the control; and weak, if there was an uncertainty and/or mild-to-moderate difference [37, 39]. The panel then re-wrote any adapted recommendation to be clear, specific and unambiguous as per GRADE [40, 41].

For each recommendation the panel drafted decision rationale, summary justifications for their judgements, detail justifications for important EtD criteria (if the recommendation was fully assessed), and considerations for implementation, special subgroups (including for geographically remote and Aboriginal and Torres Strait Islander populations), monitoring and potential future research priorities [36, 37, 39], in the Australian context [33]. The panel collated all recommendations (and rationale) into a consultaton draft manuscript of the Australian evidence-based wound classification guideline ready for public consultation [33].

The consultation draft manuscript of the wound classification guideline underwent a formal one-month public consultation period using a customised consultation survey from ADAPTE [33, 35]. All relevant survey and written feedback from the consultation period was collated, analysed and the manuscript was revised accordingly by the authors [33, 35]. Finally, the authors sought endorsement from the Australian DFD Guidelines working group and other relevant peak national bodies for the final guideline to be released. We refer the reader to the results section below for all final recommendations and rationale contained in the new Australian national evidenced-based guidelines for the wound classification in people with DFU.

## Results

The five recommendations from the IWGDF 2019 guidelines on the classification of diabetes-related foot ulcers were evaluated by the panel to determine the quality of evidence, strength of recommendation, acceptability and applicability to the Australian context.

After screening, two recommendations were adopted and three recommendations required further full assessment (see Table 1). Following full assessment by the panel, all three of those recommendations were adapted, with the adapted versions determined to be acceptable and applicable to an Australian context (see Table 2). The main reasons for adapting, were to align the recommendations with existing Australian standards of care, especially in specialist settings, such as iHRFS, to maintain consistency with existing recommendations for documentation, audit and benchmarking, and to be more appropriate, acceptable and applicable to an Australian context [42, 43]. Wording differences between the original three IWGDF and new Australian recommendations for wound classification in people with DFU are summarized in Table 3. See the Supplementary Material for detailed justification for the three recommendations that were adapted.

**Table 1:** Summary of screening ratings for acceptability and applicability in the Australian context for all IWGDF wound classification recommendations

Recommendation	Acceptability			Applicability				Full assessment	Comments
	1	2	3	4	5	6	7		
1	+	+	?	+	+	?	+	Yes	Assess acceptability, culture, values and expertise in local context
2	?	?	–	+	+	+	?	Yes	Assess strength of evidence and recommendation, culture, values
3	+	+	+	+	+	+	+	No	
4	+	+	+	+	+	+	+	No	
5	+	+	?	+	+	+	?	Yes	Assess acceptability, culture, values, local policies or constraints
Total	**4**	**4**	**2**	**5**	**5**	**4**	**3**	**3**	
%	**80%**	**80%**	**40%**	**100%**	**100%**	**80%**	**60%**	**60%**	

Note: +, yes item is met; −, no item is not met;? unsure if item is met

**Table 2:** Summary of final panel judgements compared with IWGDF judgements for all IWGDF wound classification recommendations

No	Problem	Desirable effects	Undesirable effects	Quality of evidence	Values	Balance of effects	Acceptability	Applicability/Feasibility	Decision	Comment
1	+	+	+	+	+	+	Partially agreed	Partially agreed	Adapted	Adapted acceptability & feasibility
2	+	+	+	+	+	–	–	–	Adapted	Adapted balance of effects, acceptability & feasibility
3	=	=	=	=	=	=	=	=	Adopt	Adopted in screening
4	=	=	=	=	=	=	=	=	Adopt	Adopted in screening
5	+	+	+	+	+	+	Partially agreed	Partially agreed	Adapted	Adapted acceptability & feasibility

Note: +, panel agreed with original IWGDF judgement; −, panel disagreed with original IWGDF judgement;?, panel unsure if agreed with original IWGDF judgement due to lack of IWGDF information on judgement; =, panel agreed with original IWGDF judgements during screening (see Table 1)

**Table 3:** Summary of the original IWGDF recommendations compared with the new Australian guideline recommendations for wound classification

No	Original IWGDF Recommendation	Decision	New Australian Recommendation
1	In a person with diabetes and a foot ulcer, use the SINBAD system for communication among health professionals about the characteristics of the ulcer (strong; moderate)	Adapted	In a person with diabetes and a foot ulcer, as a minimum, use the SINBAD wound classification system for communication among health professionals about the characteristics of the ulcer (strong; moderate)
2	Do not use any of the currently available classification/scoring systems to offer an individual prognosis for a person with diabetes and a foot ulcer (strong; low)	Adapted	Be cautious in the application of any of the currently available classification/scoring systems to offer an individual prognosis for a person with diabetes and a foot ulcer (weak; low)
3	In a person with diabetes and an infected foot ulcer, use the IDSA/IWGDF infection classification to characterise and guide infection management (weak; moderate)	Adopted	As stated in original IWGDF recommendation
4	In a person with diabetes and a foot ulcer who is being managed in a setting where appropriate expertise in vascular intervention is available, use WIfI scoring to aid decision making in the assessment of perfusion and likelihood of benefit from revascularisation (weak; moderate)	Adopted	As stated in original IWGDF recommendation
5	Use the SINBAD system for any regional/national/international audits to allow comparisons between institutions on the outcomes of patients with diabetes and an ulcer of the foot (strong; high)	Adapted	As a minimum, use the SINBAD system for any regional/national/international audits to allow comparisons between institutions on the outcomes of patients with diabetes and an ulcer of the foot (strong; high)

Note: underlined wording indicates the specific adapted changes to the original IWGDF recommendation

**Fig. 1:**
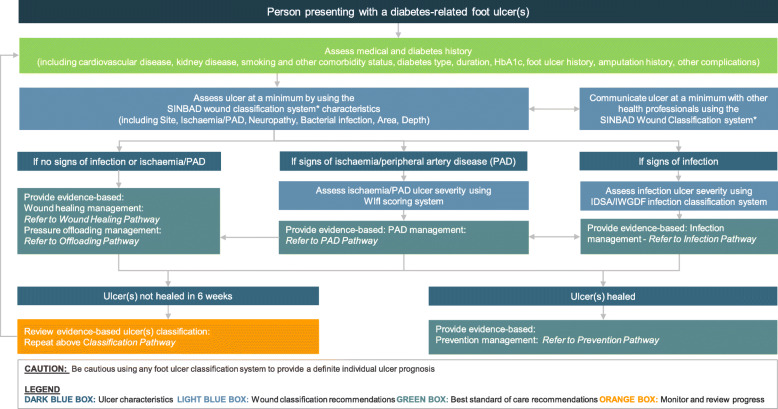
Australian evidence-based clinical pathway on wound classification of foot ulcers for people with diabetes

For each of the five Australian wound classification recommendations, we have outlined below: the question the recommendation addressed; the Australian recommendation; the panel decision and rationale to adopt, adapt or exclude; summary (and detailed if applicable) justification for the recommendation; and considerations for implementation, special subgroups (including for Aboriginal and Torres Strait Islander and geographical remote populations), monitoring; and, potential future research priorities. Following on from the Recommendations determined in this document, a consensus Clinical Pathway was developed for Wound Classification in people with DFUs, as shown in Fig. 1.

Four responses from four organisations to the public consultation survey were received. All four respondents (strongly) agreed that the guideline should be approved as the new Australian classification guideline, that the guideline would be supported by the majority of their colleagues and if approved they would encourage its use in practice. All de-identified feedback comments received during public consultation and the panel’s responses to each comment were collated and posted on the Diabetes Feet Australia website. Based on the collated public consultation feedback, the guideline was revised, approved by the panel and Australian DFD Guidelines working group, and endorsed as the new *Australian guideline on wound classification of diabetes-related foot ulcers* by ten peak national bodies including the Australian Podiatry Association, Wounds Australia, Australian and New Zealand Society for Vascular Surgery, Australasian Society for Infectious Diseases, Australian Orthotic Prosthetic Association, Pedorthic Association of Australia, Australian Advanced Practicing Podiatrists - High Risk Foot Group, Australian Aboriginal and Torres Strait Islander Diabetes-related Foot Complications Program, Australian Diabetes Society and Diabetes Feet Australia.

### Question one

#### In individuals with an active DFU, which classification system should be used in communication among health professionals to optimise referral?

##### Recommendation 1

In a person with diabetes and a foot ulcer, as a minimum, use the SINBAD wound classification system for communication among health professionals about the characteristics of the ulcer.

***Decision: Adapted.***

Rationale.

The panel decided to adapt this recommendation after full assessment, based on minor differences in some judgements to the IWGDF, particularly regarding acceptability and feasibility in an Australian context (see Table 2). As a result, wording changes to the original IWGDF recommendation were made, with the insertion of ‘as a minimum’ to indicate the use of the SINBAD wound classification system as a minimum standard for wound classification for the purposes of communication among health professionals.

***Summary justification.***

The panel agreed with the IWGDF evaluation of the strength of the evidence (moderate) and that health providers would place importance on the effective communication of information to facilitate appropriate referral and patient assessment. Although most patients are probably unaware of specific wound classification systems, it was also agreed that patients would likely place importance on effective communication of clinical information that would facilitate appropriate triage of referrals for DFU asessment and management. There were some minor differences in comparison to the IWGDF judgement for this recommendation, with partial agreement with IWGDF in regard to acceptability and feasability in an Australian context, due to existing guidelines and recommendations for use of WIfI and/ or University of Texas wound classification systems in specialist settings such as iHRFS, as well as current lack of widespread familiarity with the SINBAD wound classification system in Australia [42, 43]. The detailed justifications for our full assessment are described in Appendix 1 of the Supplementary Material.

***Implementation considerations.***

The panel agreed that the use of the additional text ‘as a minimum’ in the recommendation for the Australian Guidelines provides two additional strengths. Firstly, it recognises that SINBAD is the minimum acceptable method for wound classification, suitable for communication between health professionals, for example to and from primary care settings. Secondly, it highlights for communication between other health care providers, such as within and between iHRFS, use of an additional, more detailed wound classification system is desirable such as WIfI or University of Texas. Given the simplicity and lack of need for specialised equipment, there should be no significant barriers to implementation of the use of SINBAD in Australia. In agreement with IWGDF, it is important the individual components of SINBAD (rather than the total score) are used for the purposes of communication between health professionals. It is likely in Australia that additional educational measures will be required to support more widespread familiarity and use of SINBAD across diverse clinical settings.

***Subgroup considerations.***

Geographical remote people.

The panel agreed with the IWGDF, that SINBAD would be acceptable for use in remote locations, given the simplicity, reliability and no requirement for specialised clinical equipment.

Aboriginal and Torres Strait Islander people.

The SINBAD wound classification system would likely be well accepted and utilised in health settings where Aboriginal and Torres Strait Islander populations are managed, especially given the simplicity, reliability and no requirement for specialised clinical equipment.

Other subgroup considerations.

No other subgroup considerations.

***Monitoring considerations.***

As SINBAD is not currently widely used in Australia the panel determined that it would be useful to monitor use of SINBAD across clinical care settings in the future. This may be possible via updates inclusive of SINBAD, for the DFA minimum dataset reporting, NADC iHRFS data collection, and benchmarking or via individual primary care or hospital audits. Furthermore, the panel felt that it would be helpful to monitor how SINBAD is being used, either as a total score only, or with reporting of individual components. The effectiveness of SINBAD as a communication and triage tool depends on widespread adoption and use by health professionals across the care spectrum, so the panel felt it was important to monitor the use of SINBAD subsequent to the release of these recommendations.

***Future research considerations.***

The critical review of diabetic foot ulcer classification systems recently conducted by the IWGDF identified eight important prognostic features of a DFU, however no existing wound classification system includes all of these variables [18, 19]. In agreement with the IWGDF, future research should investigate whether the addition of more complexity to existing wound classification systems can improve clinical and prognostic utility without compromising reliability and/ or simplicity of use [19]. Furthermore, there may be uniquely Australian considerations when evaluating prognostic utility of a wound classification system in an Australian setting - Aboriginal and Torres Strait Islander people and people living in rural and remote locations experience a higher rate of LEA [9, 44] however these important patient-related factors are not included in any existing wound classification or scoring system.

As per the panel’s recommendations for monitoring of this recommendation, future research should also address the clinical uptake and usage of SINBAD in Australia across the spectrum of care settings. This may include quantitative and qualitative surveys conducted by specialist societies (e.g. RACGP, AWTRS, APP), to target groups such as general practitioners, practice nurses, nurse practitioners, orthotists/prosthetists, and podiatrists as well as via accreditation, benchmarking and reporting processes for iHRFS.

### Question two

#### In individuals with an active DFU, which classification/scoring system should be considered when assessing an individual patient to estimate their prognosis?

##### Recommendation 2

Be cautious in the application of any of the currently available classification/scoring systems to offer an individual prognosis for a person with diabetes and a foot ulcer.

***Decision: Adapted.***

Rationale.

The panel decided to adapt this recommendation after full assessment based on differences in some judgements to the IWGDF, particularly regarding overall balance of effects, acceptability and feasibility in an Australian context (see Table 2). Consequently, wording changes to the original IWGDF recommendation were made, with the insertion of the words ‘Be cautious in the application of’ instead of ‘Do not use’ to reflect the panel’s determination that prognostic information is important for patients, is useful to support clinical management, and wound classification systems are available which have been validated (albeit, at a cohort rather than individual level) for outcome prediction including DFU healing outcomes and LEA.

***Summary justification.***

The panel agreed with the IWGDF evaluation of the strength of the evidence (low) [19]. There are a number of wound classification systems that have been validated in patients with DFU for wound healing and LEA outcomes within cohorts but not at an individual patient level [18, 19]. There were some minor differences in comparison to the IWGDF judgement for this recommendation, with disagreement with IWGDF regarding overall balance of effects, acceptability and feasability in an Australian context. Acknowledging the limitations of the existing evidence, the panel agreed that cautious provision of prognostic information would be important and beneficial for both patient and clinician. As such the panel recommended caution in the use of wound classification systems such as WIfI, which is already accepted and utilised by Australian iHRFS clinicians, as well as SINBAD, as both have been found to be reliable and validated in cohorts including patients with DFU. The panel agreed it is important to consider that the term ‘prognosis’ relates not to an absolute, definitive determination but enables some determination of the overall likelihood of recovery, for example healing of a DFU by conservative management without the need for amputation. In that context, we contend that wound classification systems such as WIfI and SINBAD do provide some prognostic information for individual patients with DFU. The detailed justifications from our full assessment are described in Appendix 2 of the Supplementary Material.

***Implementation considerations.***

The panel carefully considered whether they should provide a negative recommendation, no recommendation, or a more limited positive recommendation, recognising that prognosis of diabetes-related foot ulcer healing can only be estimated and partially determined in most cases. In that context, the wording of this recommendation was modified to recognise the limitations of prognostication about a foot ulcer healing outcome at an individual patient and wound level, yet with the recognition that some data does exist (particularly for SINBAD and WIfI) to support the experienced clinician to provide a prognosis regarding DFU outcomes, including wound healing likelihood and LEA risk.

***Subgroup considerations.***

Geographical remote people.

The panel determined that this recommendation is applicable to geographically remote populations. People in geographically remote sites are at increased risk of ulcer non-healing by conservative measures and the greater likelihood of the need for LEA compared with those in urban areas. While local service factors can aid in development of data, the opinion of the panel was that overall prognosis for conservative ulcer healing is likely linked to the WIfI classification of an ulcer.

Aboriginal and Torres Strait Islander people.

The panel determined that this recommendation is applicable to Aboriginal and Torres Strait Islander people, who are at markedly increased risk of ulcer non-healing by conservative measures and the greater likelihood of the need for LEA. It is likely that overall prognosis for conservative ulcer healing and for amputation risk is linked to the WIFi scoring of an ulcer and cautious use of this information would be beneficial to Aboriginal and Torres Strait Islander people with DFU.

Other subgroup considerations.

No other subgroup considerations.

***Monitoring considerations.***

The panel felt that specific monitoring for this recommendation could include clinical practice surveys which could be systematically undertaken to determine if iHRFS in Australia in particular, utilise prognostic wound classification systems for individual patients.

***Future research considerations.***

Future research considerations for recommendation 2 are similar to those for recommendation 1. The critical review of diabetic foot ulcer classification systems recently conducted by the IWGDF identified eight important prognostic features of a DFU, however no existing wound classification system includes all of these variables [18, 19]. In agreement with the IWGDF, future research should investigate whether the addition of more complexity to existing wound classification systems can improve clinical and prognostic utility without compromising reliability and/ or simplicity of use [19]. Furthermore, there may be uniquely Australian considerations when evaluating prognostic utility of a wound classification system in an Australian setting - Aboriginal and Torres Strait Islander people and people living in rural and remote location experience a higher rate of LEA however these important patient-related factors are not included in any existing wound classification or scoring system [9, 44]. Future research work should explore the utility and reliability of existing wound classification systems such as SINBAD and WIfI for providing an individual prognosis. Furthermore, whether the addition of complexity to existing wound classifications systems via additional parameters or measures improves ability to predict outcomes without compromising reliability or utility should be the subject of further study. Finally, the development of an Australian DFU prognostic tool or score could be considered for further investigation, with the inclusion of parameters that are uniquely important to outcomes in an Australian context, such as a patient being from a geographically remote location, or from an Aboriginal or Torres Strait Islander population. Such an Australian approach would, in time, be expected to help to validate use of a prognostic tool in the domestic context.

### Question three

#### In persons with an active DFU, can any classifications/scoring system aid decision-making in specialty areas to improve healing and/or reducing amputation risk?

##### Recommendation 3

In a person with diabetes and an infected foot ulcer, use the IDSA/IWGDF infection classification to characterise and guide infection management.

***Decision: Adopted.***

Rationale.

The panel decided to adopt this recommendation after initial assessment (ADAPTE), as there was agreement amongst the panel with the IWGDF evaluation of the evidence and judgement. We agreed with the IWGDF that there are only two wound classification systems that provide assessment and stratification that can aid clinical decision making - the IDSA/IWGDF infection classification and WIfI [18, 19]. The IDSA/IWGDF infection classification systems describe four grades of DFU infection severity. Although the IDSA/IWGDF infection classification is incorporated into the foot infection component of the WIfI wound classification system, it has also been evaluated as a stand-alone classification system for diabetes-related foot ulcers complicated by infection and is used widely to help guide clinical management decisions for diabetes-related foot infections such as hospitalisation and use of intravenous antibiotics [18, 19]. The panel agreed with the IWGDF strength of evidence as ‘moderate’ given the moderate reliability, strong prediction of hospitalisation (albeit unsurprising given the clinical context of use) and validation for risk of minor and major amputation [18, 19, 45–47]. The panel also agreed with the IWGDF strength of recommendation as ‘weak’, because despite the quality of evidence, the IDSA/IWGDF infection classification is reasonably complex and has not been evaluated in diverse clinical settings [18, 19]. Finally, the panel agreed that the IDSA/IWGDF infection classification would be both acceptable and applicable in an Australian context, as there is already familiarity and widespread use in Australian healthcare settings, and no potential barriers to use in primary care or rural locations such as requirement for specialised equipment or expertise [19].

***Subgroup considerations.***

Geographical remote people.

This recommendation refers predominantly to specialty practice, however the IDSA/IWGDF infection classification would be applicable and acceptable in geographically remote locations as it is based on assessment of clinical features of infection and requires no specialised equipment.

Aboriginal and Torres Strait Islander people.

No additional considerations were identified for clinicians implementing this recommendation in regard to Aboriginal and Torres Strait Islander populations, especially as it requires no specialised equipment.

Other subgroup considerations.

No other subgroup considerations.

***Monitoring considerations.***

The panel felt there were no specific monitoring implications for this recommendation, however, advise clinicians to consider the general monitoring implications for the chapter when implementing this recommendation.

***Future research considerations.***

The panel felt there were no specific future research considerations for this recommendation, however, advise clinicians to consider the general future research considerations for the chapter when implementing this recommendation**.**

### Question four

#### In persons with an active DFU, can any classifications/scoring system aid decision-making in specialty areas to improve healing and/or reducing amputation risk?

##### Recommendation 4

In a person with diabetes and a foot ulcer who is being managed in a setting where appropriate expertise in vascular intervention is available, use WIfI scoring to aid decision-making in the assessment of perfusion and likelihood of benefit from revascularisation.

***Decision: Adopted.***

Rationale.

The panel decided to adopt this recommendation after initial assessment (ADAPTE), as there was agreement amongst the panel with the IWGDF evaluation of the evidence and judgement. WIfI generates a combined score across three areas- wound (depth of ulcer or gangrene extent), ischaemia (based on evaluation with ankle-brachial index, ankle systolic pressure, toe pressure or transcutaneous oxygen pressure) and foot infection (IDSA/IWGDF infection classification) and can be used to stratify one year risk of amputation and one year benefit from revascularisation, which are both classified as very low, low, moderate or high [6, 18, 19]. The panel agreed with the IWGDF evaluation of evidence as ‘moderate’ given the strong evidence of reliability for outcome prediction, including wound healing, need for revascularisation and LEA in patient cohorts with peripheral arterial disease, but less so specifically in patient cohorts with DFU [48–52]. The panel also agreed with the IWGDF strength of recommendation as ‘weak’, because despite the quality of evidence, the WIfI classification is reasonably complex and has not been evaluated in diverse clinical settings and populations [18, 19]. Finally, the panel agreed that the use of WIfI classification system to aid clinical decision making and evaluate benefit from revascularisation in clinical contexts where appropriate vascular surgical expertise is available would be both acceptable and applicable in an Australian context, as there is already familiarity and widespread acceptance of use in Australian guidelines and recommendations, particularly in the setting of iHRFS, in which the presence of Vascular surgical expertise is recommended [42, 43].

***Subgroup considerations.***

Geographical remote people.

This recommendation refers predominantly to specialty practice where vascular surgeon expertise is available. As such, this recommendation is not generally applicable to geographically remote locations as it requires specialist expertise and equipment.

Aboriginal and Torres Strait Islander people.

When assessment is being made, and if revascularisation is being considered, there must be adequate consultation with the patient and engagement with family explaining why the assessment is being conducted and if hospitilisation is needed provide the approximate length of stay required. There must also be consideration of language barriers with consultation, especially where English may be a second, third or fourth language, in these situations a professional interpreter should be considered.

Other subgroup considerations.

No other subgroup considerations.

***Monitoring considerations.***

The panel felt there were no specific monitoring implications for this recommendation.

***Future research considerations.***

The panel felt there were no specific future research considerations for this recommendation.

### Question five

#### In persons with an active DFU, which classification/scoring system should be considered for regional/national/international audit to allow comparisons between institutions?

##### Recommendation 5

As a minimum, use the SINBAD system for any regional/national/international audits to allow comparisons between institutions on the outcomes of patients with diabetes and an ulcer of the foot.

***Decision: Adapted.***

Rationale.

The panel decided to adapt this recommendation after full assessment based on minor differences in some judgements to the IWGDF, particularly regarding acceptability and feasibility in an Australian context (see Table 2). As a result, wording changes to the original IWGDF recommendation were made, with the insertion of ‘as a minimum’ to recommend the use of the SINBAD wound classification system as a minimum standard for regional/national/international audits to allow comparisons between institutions.

***Summary justification.***

The panel agreed with the IWGDF evaluation of the strength of the evidence (strong) and that health providers would place importance on the reliability of wound classification systems used for the purposes of regional, national and international audit and benchmarking but also on the simplicity and ease of use of such a system across diverse populations and care settings. There was some minor differences in comparison to the IWGDF judgement for this recommendation, with partial agreement with IWGDF regarding acceptability and feasability in an Australian context, due to existing guidelines and recommendations for use of WIfI and/ or University of Texas wound classification systems for audit and benchmarking in specialist settings such as iHRFS, as well as current lack of widespread familiarity with the SINBAD wound classification system in Australia [42, 43]. Use of the additional text ‘As a minimum’ in the recommendation for the Australian Guidelines provides two additional strengths. Firstly, it recognises that the use of SINBAD for wound classification reporting for the purpose of audit is the minimum acceptable method and would be acceptable and appropriate in settings such as primary care. Secondly, it recognises that in other health care settings such as iHRFS, additional information provided by a more detailed wound classification system such as WIfI or University of Texas would be desirable, and is recommended in Australian standards of care [42, 43]. The additional text does not negate that the SINBAD scoring system is reliable, appropriate and its use for the purpose of audit is supported by evidence, but it does indicate that it may be insufficient in some clinical settings, particularly in specialist iHRFS care. The detailed justifications from our full assessment are provided in Appendix 3 of the Supplementary Material.

***Implementation considerations.***

The panel agreed that the use of the additional text ‘as a minimum’ in this recommendation provides two additional strengths. Firstly it recognises that SINBAD is the minimum acceptable standard for wound classification for the purposes of regional/ national/ international audit and should be completed for all patients with DFU. Secondly, it highlights for certain care settings, such as iHRFS, use of an additional, more detailed wound classification system is desirable such as WIfI or University of Texas to appropriately capture DFU characteristics to enable more accurate audit and benchmarking. Given the simplicity of SINBAD and lack of need for specialised equipment, there should be no significant barriers to implementation of use of SINBAD in Australia. In agreement with IWGDF, it is important the individual components of SINBAD (rather than the total score) are used for the purposes of audit [19]. It is likely in Australia that additional educational measures will be required to support more widespread familiarity and use of SINBAD across diverse clinical settings.

***Subgroup considerations.***

Geographical remote people.

The panel determined that this recommendation is applicable to geographically remote populations. Given the simplicity, reliability and ease of use, the use of SINBAD for audit purposes was thought likely to be accepted and readily utilised in rural and regional health care settings.

Aboriginal and Torres Strait Islander people.

The panel determined that this recommendation is applicable to Aboriginal and Torres Strait Islander people. The SINBAD wound classification system would likely be well accepted and utilised in health settings where Aboriginal and Torres Strait Islander populations are managed.

Other subgroup considerations.

No other subgroup considerations.

***Monitoring considerations.***

SINBAD is not currently widely used in Australia and consequently, the panel determined that it would be useful to monitor the use of SINBAD for audit purposes across clinical care settings in the future. This may be possible via DFA minimum dataset reporting, NADC iHRFS data collection and benchmarking or via individual primary care or hospital audits. Furthermore, the panel felt that it would be helpful to monitor how SINBAD is being used, either as a total score or reporting of individual components. The effectiveness of SINBAD as an audit tool in an Australian context depends on widespread adoption and use by health professionals across the care spectrum, so the panel felt it was important to monitor the use of SINBAD subsequent to the release of these recommendations.

***Future research considerations.***

Future research considerations for recommendation 5 are similar to those for recommendation 1. The critical review of diabetic foot ulcer classification systems recently conducted by the IWGDF identified eight important prognostic features of a DFU, however no existing wound classification system includes all of these variables [18, 19]. In agreement with the IWGDF, future research should investigate whether the addition of more complexity to existing wound classification systems can improve clinical and prognostic utility without compromising reliability and/ or simplicity of use [19]. Furthermore, there may be uniquely Australian considerations when evaluating prognostic utility of a wound classification system in an Australian setting- Aboriginal and Torres Strait Islander people and people living in rural and remote location experience a higher rate of LEA [9, 44], however these important patient-related factors are not included in any existing wound classification or scoring system.

As per the panel’s recommendations for monitoring of this recommendation, future research should also address the clinical uptake and usage of SINBAD for the purpose of audit in Australia across the spectrum of care settings. In addition, it is recognised that reporting of the individual components of SINBAD separately for a foot ulcer in a person with diabetes, adds detailed clinical value compared with the SINBAD score alone. Thus the monitoring systems above and the qualitative research targeting primary care could also determine how often the individual components of SINBAD are reported, in addition to the score out of six. This may include qualitative surveys conducted by specialist societies (e.g. RACGP, AWTRS, APP) to target groups such as general practitioners, practice nurses, nurse practitioners, and podiatrists as well as via accreditation, benchmarking and reporting processes for iHRFS.

## Discussion

### Recommendations summary

The classification of DFUs is central to achieving optimal outcomes for people with diabetes and is important to facilitate effective communication among health professionals, timely triage and assessment, to guide management decisions and prognosis, and to support audit and benchmarking activities. After the five recommendations from the IWGDF 2019 guidelines on the classification of diabetic foot ulcers were evaluated by the panel, two were adopted and three were adapted to be more suitable for Australian conditions. The main reasons for adapting, were to align the recommendations to existing Australian standards of care, especially in specialist settings, to maintain consistency with existing Australian recommendations for documentation, audit and benchmarking and to be more appropriate, acceptable and applicable to an Australian context. In Australia, we recommend the use of the SINBAD system as a minimum standard to document the characteristics of a DFU for the purposes of communication among health professionals and for regional/ national/ international audit. We have adopted the 2019 IWGDF recommendations for the use of the IDSA/IWGDF infection classification system to characterise and guide management of an infected DFU in a person with diabetes and the WIfI classification system to guide perfusion assessment and benefit from revascularisation in settings where vascular surgical expertise is available. In contrast to the IWGDF who make a recommendation against usage, in Australia we recommend caution in the use of existing wound classification systems to provide a prognosis for a person with diabetes and a foot ulcer.

### Justifications summary

There were some minor differences in comparison with the IWGDF judgement for recommendations 1 and 5, with partial agreement with IWGDF in regard to acceptability and feasability in an Australian context, due to existing guidelines and recommendations for use of WIfI and/ or University of Texas wound classification systems for communication among health professionals and for audit and benchmarking in specialist settings such as iHRFS, as well as current lack of widespread familiarity with the SINBAD wound classification system in Australia [42, 43]. Use of the additional text ‘As a minimum’ in both recommendation 1 and 5 for the Australian Guidelines provides two additional strengths. Firstly, it recognises that the use of SINBAD for wound classification reporting for the purpose of communication and audit is the minimum standard and would be acceptable and appropriate in settings such as primary care. Secondly, it recognises that in other health care settings such as iHRFS, additional information provided by a more detailed wound classification system such as WIfI or University of Texas would be desirable, and is recommended in Australian standards of care [42, 43]. There were some differences in comparison with the IWGDF judgement for Recommendation 2, with disagreement with IWGDF regarding the overall balance of effects, and acceptability and feasability in an Australian context. Acknowledging the limitations of the existing evidence, the panel agreed that cautious provision of prognostic information would be important and beneficial for both patient and clinician. As such the panel recommended cautious use of wound classification systems such as WIfI, which is already accepted and utilised by Australian iHRFS clinicians, as well as SINBAD, as both have been found to be reliable and validated in cohorts including patients with DFU .

### Subgroup considerations summary

In general, the new Australian Recommendations for wound classification in people with diabetes and a foot ulcer were not associated with specific concerns or considerations for the management of DFU in Aboriginal and Torres Strait Islander people or for people living in geographically remote locations. In particular, the panel felt that the SINBAD wound classification system would likely be well accepted and utilised in health settings where Aboriginal and Torres Strait Islander populations are managed.

In addition, the panel agreed with the IWGDF, that the SINBAD system would be acceptable for use in remote locations, given the simplicity, reliability and no requirement for specialised clinical equipment. There were no other significant subgroup considerations applicable to the wound classification recommendations.

### Implementation considerations summary

Given the simplicity and lack of need for specialised equipment, there should be no significant barriers to implementation of use of SINBAD in Australia for the purpose of communication among health professionals and/ or regional/ national/ international audit (recommendations 1 and 5). In agreement with the IWGDF, it is important the individual components of SINBAD (rather than the total score) are used for the purposes of communication between health professionals. It is likely in Australia that additional educational measures will be required to support more widespread familiarity and use of SINBAD across diverse clinical settings. In regard to recommendation 2, there are likely no specific implementation considerations as the use of WIfI is already established in Australian iHRFS care standards [42, 43].

### Monitoring considerations summary

SINBAD is not currently widely used in Australia and as such, the panel determined that it would be useful to monitor adoption of SINBAD across clinical care settings in the future. This may be possible via DFA minimum dataset reporting, NADC iHRFS data collection and benchmarking or via individual primary care or hospital audits. Furthermore, the panel felt that it would be helpful to monitor how SINBAD is being used, either as a total score or reporting of individual components. The effectiveness of SINBAD as a communication and audit tool depends on widespread adoption and use by health professionals across the care spectrum, so the panel felt it was important to monitor the use of SINBAD subsequent to the release of these recommendations. In regard to the use of wound classifications systems such as SINBAD and WIfI to provide an individual prognosis, the panel felt that specific monitoring could include clinical practice surveys to determine if iHRFS in Australia utilise prognostic wound classification systems for individual patients.

### Future research considerations summary

The critical review of diabetic foot ulcer classification systems recently conducted by the IWGDF identified eight important prognostic features of a DFU, however no existing wound classification system includes all of these variables [18, 19]. In agreement with the IWGDF, future research should investigate whether the addition of more complexity to existing wound classification systems can improve clinical and prognostic utility without compromising reliability and/ or simplicity of use [19]. Furthermore, there may be uniquely Australian considerations when evaluating prognostic utility of a wound classification system in an Australian setting - Aboriginal and Torres Strait Islander people and people living in rural and remote location experience a higher rate of LEA however these important patient-related factors are not included in any existing wound classification or scoring system [9, 44]. Future research work should also explore the utility and reliability of existing wound classification systems such as SINBAD and WIfI for providing an individual prognosis. Furthermore, whether the addition of complexity to existing wound classifications systems via additional parameters or measures improves ability to predict outcomes without compromising reliability or utility should be the subject of further study. Finally, the development of an Australian DFU prognostic tool or score could be considered for further investigation, with the inclusion of parameters that are uniquely important to outcomes in an Australian context, such as a patient being from a geographically remote location or from an Aboriginal or Torres Strait Islander population.

## Conclusion

We have developed new guidelines for wound classification for people with DFU that are appropriate and applicable for use across diverse care settings and geographical locations in Australia, including Aboriginal and Torres Strait Islander populations and people living in remote locations. These recommendations should serve to support clinicians to provide more effective communication, reliable prognostication and conduct detailed and productive audit and benchmarking activities in an Australian context. Whilst the panel agrees with the IWGDF that it is possible that no single wound classification system will be developed for DFU that is suitable for all clinical scenarios, future research should focus on whether the addition of more parameters and complexity to existing DFU wound classification systems results in provision of a more reliable estimation of key DFU outcomes.

## Supplementary Information

**Additional file 1.**

## Data Availability

Data sharing is not applicable to this article as no datasets containing patient information were generated or analysed during the current study.

## Abbreviations

DFA: Diabetes Feet Australia
DFD: Diabetes-related foot disease
DFU: Diabetes-related foot ulcer
EtD: Evidence to Decision
GRADE: Grading of Recommendations Assessment, Development and Evaluation
IDSA: Infectious Diseases Society of America
iHRFS: interdisciplinary high-risk foot service
IWGDF: International Working Group on the Diabetic Foot
LEA: Lower extremity amputation
NADC: National Association of Diabetes Centres
NHMRC: National Health and Medical Research Council
RACGP: Royal Australian College of General Practitioners
SINBAD: Site, Ischaemia, Neuropathy, Bacterial Infection, Area and Depth
WIfi: Wound, Ischaemia, and foot Infection
