# Supplementary material for: Australian guideline on wound classification of diabetes-related foot ulcers: part of the 2021 Australian evidence-based guidelines for diabetes-related foot disease
Source: J Foot Ankle Res. 2021 Dec 3;14:60. doi: 10.1186/s13047-021-00503-6 (PMC8641146; doi:10.1186/s13047-021-00503-6)
Supplement: Supplementary file 1 — Additional file 1. [file 13047_2021_503_MOESM1_ESM.pdf]

Online-Only Supplementary Material for:

Australian guideline on wound classification of diabetes-related foot ulcers: Part of the 2021 Australian evidence-based guidelines for diabetes-related foot disease

Hamilton EJ, Scheepers J, Ryan H, Perrin B, Charles J, Cheney J, Twigg SM, on behalf of the Australian Diabetes-related Foot Disease Guidelines & Pathways Project

**Table of contents**	**Page**
**Appendix 1:** Detailed justifications for Recommendation 1	2
**Appendix 2:** Detailed justifications for Recommendation 2	5
**Appendix 3:** Detailed justifications for Recommendation 5	6
**References**	8

Appendix 1: Detailed justifications for Recommendation 1

In a person with diabetes and a foot ulcer, as a minimum, use the SINBAD wound classification system for communication among health professionals about the characteristics of the ulcer (GRADE strength of recommendation: Strong; Quality of evidence: Moderate).

Problem: Agreed. The panel agreed with the IWGDF (1) that the use of a classification system to communicate information clearly and effectively about the characteristics of a DFU was of high importance, to aid appropriate triage of referrals, communication of information between practitioners, continuity of care, as well as quality assurance activities.

Desirable effects: Agreed. The panel agreed it is highly desirable to have as a minimum, one validated wound classification system for communication amongst health professionals, for a DFU in a person with diabetes. The panel agreed with the IWGDF, that for a wound classification system to be used by all health professionals involved in the care of people with DFU, it needs to be quick and simple to use, require no specialised equipment or expertise, but also communicate important information about the DFU to facilitate accurate triage and timely intervention by the receiving practitioner (1). The panel also agreed with the IWGDF that although ideally all patients with a DFU should be referred and reviewed by an iHRFS as a matter of urgency, it was important that a classification system included critical information that would necessitate urgent review, including size of the ulcer (area and depth), presence of infection and presence of ischaemia, ideally without requiring specialist equipment (1). There are a number of classification systems which have been validated for ulcer healing and lower extremity amputation occurrence, including Meggit-Wagner, SINBAD, University of Texas and WIfI, however of these systems, only SINBAD includes the necessary information for triage, while also being quick, simple and requiring no specialist equipment (1). The SINBAD system grades six DFU characteristics, including site, ischaemia, neuropathy, bacterial infection, area and depth as either zero or one point, with a maximum of six points (2).

Of the available wound classification systems, the panel felt that the SINBAD system had a number of advantages. The panel agreed with the IWGDF that SINBAD is a simple system, requiring clinical assessment alone, and yet it includes six key parameters for describing a DFU. It has also been shown to have good reliability, both intra- and inter- observer. All of these considerations mean that it would be a good communication tool particularly between an iHRFS and primary care.

There are some significant challenges and concerns however in prospectively recommending using SINBAD as the only, single recommended wound classification system in Australia for wounds in diabetes. Firstly, SINBAD does not detail the status of the wound within each of the key aspects of infection, ischaemia, and depth. Specifically, it does not report the severity of infection, the severity of the ischaemia, nor the depth of the ulcer in any nuanced way. In comparison, other validated wound classification systems recommended for use by iHRFS and datasets in Australia do report severity, such as WIfI. Secondly, while the SINBAD wound classification system provides a simplistic scoring system, it is recognised that the global score out of six, treats each of the parameters measured as the same score of zero or one; as such a score of three out of six could have no ischaemia, no infection present nor any depth, or it could have each of those clinically

important factors. The IWGDF recognises that not only should the SINBAD score be reported for a wound but the actual individual components that are positive should also be described. Such requirement reflects that a single score using SINBAD is not optimal, particularly for clinical settings where more detail about the wound is required for management. Indeed, the IWGDF recognises that there is not likely to be one wound classification system that will meet all needs.

Thirdly, in Australia the SINBAD system of wound classification is not in current routine use by either primary care or iHRFS practitioners. While such a situation should not prevent its commencement and recommendation per se, education and familiarisation programs may be required, both for primary care and iHRFS practitioners to encourage widespread use and adoption of SINBAD in clinical care.

Undesirable effects: Agreed. The Panel recognised that it is highly desirable to have as a minimum, one validated wound classification system for communication amongst health professionals, for a DFU in a person with diabetes. The undesirable aspects of this outcome are small, however it is possible that if SINBAD is adopted and the score from six is used, rather than also reporting the individual components, it could potentially result in false reassurance. Overall, the panel determined the risks of such undesirable outcomes as being relatively small.

Quality (or certainty) of evidence: Agreed. The panel agreed with the IWGDF evaluation of the quality of evidence to support the use of SINBAD (moderate) (1, 3). The SINBAD system is in widespread use in the UK and is utilised in the UK National Diabetic Foot Audit (4). The SINBAD system is reliable and has been validated in a number of countries for both ulcer healing and lower extremity amputation (LEA) prediction in cohort studies (2, 4-10).

Values: Agreed. The panel felt that most patients were likely unaware of the wound classification systems being used by clinicians at present. However, it was agreed that patients would likely place importance on clear, effective and reliable communication of important clinical information to facilitate triage and timely review.

Balance of effects: Agreed. The panel agreed with the IWGDF that the balance of effects favoured the use of SINBAD as a single, minimum standard for wound classification in patients with DFU (1), and the desirable effects outweighed the undesirable effects.

Acceptability: Partially agreed. The panel was in partial agreement with the IWGDF. The SINBAD system is in widespread use in the United Kingdom and is the accepted compulsory standard (4). Given similarities in health care delivery between the United Kingdom and Australia, the panel agreed that SINBAD would also likely be acceptable to Australian clinicians. However, in an Australian setting, it is recommended for patients with DFU to be managed in an iHRFS, where the National Association of Diabetes Centres (NADC) iHRFS standards and Diabetic Foot Australia Minimum Dataset Dictionary recommend the use of more detailed classification systems such as WIfI and/or University of Texas (11, 12). As a result, both primary care providers and specialist iHRFS clinicians may be more familiar with wound classification systems other than SINBAD, however the panel did not think this would be a barrier in the longer term to more widespread use of SINBAD, although some education initiatives may be required initially to encourage adoption of SINBAD in Australia.

Feasiblity: Partially agreed. The panel partially agreed with the IWGDF as the simplicity and reliability of SINBAD would make it feasible for widespread clinical use as the minimum

standard for wound classification in patients with DFU in Australia. However the panel felt that SINBAD would not be the optimal wound classification system for assessment of patients with DFU in specialist clinical settings, such as iHRFS, where more detailed, nuanced wound classification systems would ideally be used, in line with recent and current Australian NADC iHRFS Standards processes, and Diabetes Feet Australia (DFA) and NADC minimal datasets (11, 12).

Appendix 2: Detailed justifications for Recommendation 2

Be cautious in the application of any of the currently available classification/scoring systems to offer an individual prognosis for a person with diabetes and a foot ulcer (weak; low)

Problem: Agreed. The panel agreed with the IWGDF (1) that the use of a classification system to provide prognostic information regarding wound healing and/ or LEA risk for a patient with a DFU was of high importance.

Desirable effects: Agreed. The panel recognised that it is highly desirable for both patients and clinicians to have available a wound classification system or risk prediction tools that can reliably predict prognosis including DFU healing outcomes and LEA risk at an individual level.

Undesirable effects: Agreed. The panel agreed that undesirable effects were likely moderate with uncertain risk associated with providing inaccurate prognostic information via the use of a wound classification system not validated at an individual patient level. However, the panel also agreed that currently in Australia, classification systems, particularly WifI which is recommended for use in iHRFS, were not utilised as a single definitive indicator of prognosis but rather as a guide to prognosis as part of a holistic assessment and management plan.

Quality (or certainty) of evidence: Agreed. The panel agreed with the IWGDF evaluation of the evidence (low) that there is no one DFU classification system that includes all eight key clinical parameters, is simple and reliable and has been validated not only in cohort studies but for individual patient outcomes (1, 3).

Values: Agreed. Consumer representatives indicated no strong preference about the use of a particular wound classification tool for an individual prognosis, recognising that, as able, evidence-based, reliable information should be shared with patients with diabetes who have foot wounds, in a balanced manner. The panel agreed that consumers may vary as to the degree to which each individual would seek and expect to receive a reliable prognosis, however having some indication of prognosis was felt to be desirable for the consumer.

Balance of effects: Disagreed. The panel disagreed with the IWGDF and determined that where possible, it is desirable to use a wound classification system, either WIfI or SINBAD, to provide prognostic information to patients with DFU regarding outcomes such as wound healing and LEA risk, however this information should be provided with caution, given the limitations of validation of risk prediction at an individual patient level.

Acceptability: Disagreed. The panel disagreed with the IWGDF and determined that it would likely be unacceptable to both patients and providers to not provide a prognosis related to DFU outcomes. Instead, with caution, an individual prognosis for DFU could be provided, based on wound classification/scoring systems such as WIfI and SINBAD that are reliable and validated, albeit at cohort rather than individual patient level.

Feasiblity: Disagreed. The panel determined that it would be feasible to use existing, validated wound classification systems, particularly WIfI and SINBAD, with caution, to predict DFU outcomes such as healing and LEA.

Appendix 3: Detailed justifications for Recommendation 5

As a minimum, use the SINBAD system for any regional/national/international audits to allow comparisons between institutions on the outcomes of patients with diabetes and an ulcer of the foot (strong; high)

Problem: Agreed. The panel agreed with the IWGDF (1) that the use of a classification system to document information effectively about the characteristics of a DFU for the purpose of audit, was of high importance, to aid comparison of outcomes for patients with DFU across health services, regions, or countries, to support benchmarking activities and to help drive improvement of service delivery and long term outcomes for people living with DFU. The panel agreed with the IWGDF that the ideal classifications system for audit should be simple, reliable, validated and require no specialised equipment in order to allow audit and comparisons across diverse populations and geographical locations.

Desirable effects: Agreed. The panel agreed with IWGDF and recognised that it is highly desirable to have as a minimum, one validated wound classification system for documenting the characteristics of a DFU in a person with diabetes, for the purpose of regional/ national/ international audit. It was recognised by the panel that in clinical settings such as primary care, there is currently no recognised wound classification system used consistently or routinely in Australia to enable routine health audit outcomes. This includes rural and regional settings and also services focused on health care delivery for Aboriginal and Torres Strait Islander people with diabetes who have foot ulcers. However, the iHRFS to which a patient will commonly be referred will be using a more complex system, and auditing the same wound classification or scoring to primary care will not likely provide additional value. In that context, routine use of the SINBAD wound classification and scoring system in Australia for audit would be a major advance upon current practice. However, in some settings using SINBAD alone would not be adequate for audit of case mix and comparison of outcomes.

Undesirable effects: Agreed. The panel recognised that it is highly desirable to have as a minimum, one validated DFU wound classification system for the purpose of audit and the undesirable aspects of this outcome are small. If SINBAD is adopted and the score from six is used rather than the individual components, it is possible that the scoring system may provide somewhat misleading information or classification, given that it is likely some SINBAD components have a greater impact on clinical outcomes than others (4). Overall, the risks of such undesirable outcomes were assessed by the panel as being relatively small.

Quality (or certainty) of evidence: Agreed. The panel agreed with the IWGDF evaluation of the quality of evidence to support the use of SINBAD for the purpose of regional/ national/ international audit (strong) (1, 3). The panel agreed with the IWGDF that to be appropriate for use for international audit, diverse clinical settings and suitable for comparison of outcomes for large numbers of patients, a DFU classification/ scoring system needs to simple, reliable, quick to perform and require no specialised equipment or expertise. The SINBAD system meets these criteria, is reliable and has been validated in a number of countries for both ulcer healing and lower extremity amputation (LEA) prediction in cohort studies (2, 4-10). The SINBAD system is in widespread use in the UK and is utilised in the UK National Diabetic Foot Audit with reporting of outcomes for over 20,000 people with DFU (4).

Values: Agreed. The panel felt that most patients were likely to be unaware of the wound classification systems being used by clinicians for the purpose of audit at present and would probably not have a specific preference. However, it was recognised by the panel that patients would likely view it as being desirable that audits report reliably and accurately about their wound and outcomes in order to enable benchmarking of health care systems and services that provide care to people with diabetes and foot ulcers. In contrast, some health professionals in Australia may prefer to, in addition to SINBAD, use detailed, validated systems which they have routinely used previously for the purpose of audit such as WIfI and University of Texas, and which are currently recommended in existing standards of care for iHRFS in Australia (11, 12).

Balance of effects: Agreed. The panel agreed with the IWGDF that the balance of effects favoured the use of SINBAD as a single, minimum standard for the purposes of regional/ national/ international audit in patients with DFU (1), and the desirable effects outweighed the undesirable effects.

Acceptability: Partially agreed. The panel was in partial agreement with the IWGDF. The SINBAD system is in widespread usage in the United Kingdom and is the accepted compulsory standard (4). Given similarities in health care delivery between the United Kingdom and Australia, the panel agreed that SINBAD would also likely be acceptable to Australian clinicians for the purpose of audit. However, the panel also recognised that to acquire a more detailed assessment of a wound, it is not optimal to indicate whether as a dichotomous variable, a wound has infection, ischaemia, or is deep or not. In an Australian context, it is recommended for patients with DFUs to be managed in an iHRFS, where the NADC iHRFS guidelines and the Diabetic Foot Australia minimum dataset dictionary recommend the use of more detailed classification systems such as University of Texas and/ or WIfI (11, 12). As a result, both primary care providers and specialist iHRFS providers may be more familiar with wound classification systems other than SINBAD, however the panel did not think this would be a barrier in the longer term to more widespread use of SINBAD for the purposes of audit, although some education initiatives may be required initially to encourage adoption of SINBAD more widely in Australia.

Feasibility: Partially agreed. The panel partially agreed with the IWGDF as the simplicity, reliability and validation of SINBAD would make it feasible for widespread use as the minimum standard for wound classification for the purpose of audit in patients with DFU in Australia. However, the panel felt that SINBAD would not be the optimal wound classification system for audit purposes in specialist clinical settings, such as iHRFS, where more detailed nuanced wound classification systems would ideally be used, in line with current Australian recommendations. When the use of SINBAD as a wound classification system comes into more routine clinical practice in Australia, it would likely be accepted as the minimum wound classification system for use in a clinical setting for audit. Being simple and reliable, the panel agreed the use of SINBAD for audit would be sustainable and suitable across diverse Australian clinical settings.

References

1. Monteiro-Soares M, Russell D, Boyko EJ, Jeffcoate W, Mills JL, Morbach S, et al. Guidelines on the classification of diabetic foot ulcers (IWGDF 2019). Diabetes Metab Res Rev. 2020;36 Suppl 1:e3273.
2. Ince P, Abbas ZG, Lutale JK, Basit A, Ali SM, Chohan F, et al. Use of the SINBAD classification system and score in comparing outcome of foot ulcer management on three continents. Diabetes Care. 2008;31(5):964-7.
3. Monteiro-Soares M, Boyko EJ, Jeffcoate W, Mills JL, Russell D, Morbach S, et al. Diabetic foot ulcer classifications: A critical review. Diabetes Metab Res Rev. 2020;36 Suppl 1:e3272.
4. Digital N. National Diabetes Foot Care Audit, 2014- 2018. 2019.
5. Forsythe RO, Ozdemir BA, Chemla ES, Jones KG, Hinchliffe RJ. Interobserver Reliability of Three Validated Scoring Systems in the Assessment of Diabetic Foot Ulcers. Int J Low Extrem Wounds. 2016;15(3):213-9.
6. Abbas ZG, Lutale JK, Game FL, Jeffcoate WJ. Comparison of four systems of classification of diabetic foot ulcers in Tanzania. Diabet Med. 2008;25(2):134-7.
7. Chuan F, Tang K, Jiang P, Zhou B, He X. Reliability and validity of the perfusion, extent, depth, infection and sensation (PEDIS) classification system and score in patients with diabetic foot ulcer. PLoS One. 2015;10(4):e0124739.
8. Jeon BJ, Choi HJ, Kang JS, Tak MS, Park ES. Comparison of five systems of classification of diabetic foot ulcers and predictive factors for amputation. Int Wound J. 2017;14(3):537-45.
9. Monteiro-Soares M, Martins-Mendes D, Vaz-Carneiro A, Dinis-Ribeiro M. Lower-limb amputation following foot ulcers in patients with diabetes: classification systems, external validation and comparative analysis. Diabetes Metab Res Rev. 2015;31(5):515-29.
10. Parisi MC, Zantut-Wittmann DE, Pavin EJ, Machado H, Nery M, Jeffcoate WJ. Comparison of three systems of classification in predicting the outcome of diabetic foot ulcers in a Brazilian population. Eur J Endocrinol. 2008;159(4):417-22.
11. Society NAoDCatAD. NADC Collaborative Interdisciplinary Diabetes High Risk Foot Services (HRFS) standards. Sydney2018.
12. Australia DF. Australian Diabetic Foot Ulcer Minimum Dataset Dictionary. Brisbane2016.
